# Reference measures of lower-limb joint range of motion, muscle strength, and selective voluntary motor control of typically developing children aged 5–17 years

**DOI:** 10.1177/18632521241234768

**Published:** 2024-05-03

**Authors:** Emily Scherff, Sabrina Elisabeth Schnell, Tobias Siebert, Sonia D’Souza

**Affiliations:** 1 Motion and Exercise Science, University of Stuttgart, Stuttgart, Germany; 2 Department of Sport and Sport Science, University of Freiburg, Freiburg, Germany; 3 Gait Laboratory, Orthopaedic Clinic, Olga Hospital, Klinikum Stuttgart, Stuttgart, Germany

**Keywords:** Typically developing, normative, range of motion, muscle strength, selective voluntary motor control

## Abstract

**Background:**

Joint range of motion based on the neutral null method, muscle strength based on manual muscle testing, and selective voluntary motor control based on selective control assessment of the lower extremity are standard parameters of a pediatric three-dimensional clinical gait analysis. Lower-limb reference data of children are necessary to identify and quantify abnormalities, but these are limited and when present restricted to specific joints or muscles.

**Methods:**

This is the first study that encompasses the aforementioned parameters from a single group of 34 typically developing children aged 5–17 years. Left and right values were averaged for each participant, and then the mean and standard deviation calculated for the entire sample. The data set was tested for statistical significance (*p* < 0.05).

**Results:**

Joint angle reference values are mostly consistent with previously published standards, although there is a large variability in the existing literature. All muscle strength distributions, except for M. quadriceps femoris, differ significantly from the maximum value of 5. The mean number of repetitions of heel-rise test is 12 ± 5. Selective voluntary motor control shows that all distributions, except for M. quadriceps femoris, differ significantly from the maximum value of 2.

**Conclusion:**

Since typically developing children do not match expectations and reference values from the available literature and clinical use, this study emphasizes the importance of normative data. Excessively high expectations lead to typically developing children being falsely underestimated and affected children being rated too low. This is of great relevance for therapists and clinicians.

**Level of evidence:**

3.

## Introduction

Passive range of motion (ROM) of joints, muscle strength, and selective voluntary motor control (SVMC) are standard parameters when assessing motor performance and gait capacity in children with motor deficits stemming from neurological or orthopedic diseases.^1^ It is important to establish reference data so that abnormalities can be identified and quantified, progression monitored, and outcomes classified.^2^

Studies have been published that provide the passive joint ROM in children and adults.^3–11^ The methods of measurements, however, vary from different types of goniometers to camera-based systems. There are other limitations such as a small sample size,^6^ a focus on only one or few joints^6–10^, or on a specific group of children.^11^

Macfarlane et al.^12^ provide strength reference data for hip and knee in 6- to 8-year-old children using a handheld dynamometer. McKay et al.^5^ established reference values for flexibility and isometric strength of ankle, knee, hip, elbow, and shoulder musculature using handheld and fixed dynamometry.

However, isometric strength testing in children with neuro-orthopedic disorders is not a feasible option. Instead, manual muscle testing (MMT) is commonly used in clinical examinations to assess and quantify muscle strength.^12^

Florence et al.^13^ observed reliability and inter-rater reliability of the grades of a modified Medical Research Council (mMRC) scale. The clinical use of the mMRC scale in manual muscle strength measurements is reported by Paternostro-Sluga et al.^14^ who recommended this method for diagnostics in peripheral nerve palsy due to the strong validity and reliability. Plantarflexor strength is difficult to assess with MMT and therefore assessed using the functional unilateral heel-rise test (HRT).^15^ Here, 20 repetitions are the expected norm.^15,16^ However, a study involving healthy adults showed that the number of repetitions was age dependent.^17^ It poses the question of whether 20 repetitions are an appropriate standard for the HRT in children.

SVMC describes the ability to perform isolated joint movements, which is difficult in people with neurological conditions due to temporal failure in muscle recruitment or to a synergistic movement pattern.^18^ SVMC is often rated based on the “Selective Control Assessment of the Lower Extremity” (SCALE) where the grade 2 is the expected standard for a normal SVMC in typically developing participants.^18^ Validity and inter-rater reliability of the methodology has been tested using children with spastic cerebral palsy (CP).^18^ There is insufficient research regarding reference values of SVMC for children and adults. Fahr et al.^19^ recognize that adults perform movements more accurately with fewer involuntary movements compared to children. This raises the question of whether the expected standard can be applied to children.

Although existing literature provides reference values of ROM and muscle strength in adults, normative data of children are limited and when present, restricted to single measurements. We are not aware of any study that sufficiently summarizes ROM, MMT, and SVMC reference values for children of the lower body. Can we expect that typically developing children exhibit muscle strengths of 5 and SVMC of 2 (isolated)? What is the variability in these measures per muscle group? These are the questions that this study aims to answer by measuring ROM, muscle strength, and SVMC in typically developing children aged 5–17 years. A normative lower body data set will be created to provide a full reference data set for a clinical pediatric examination as part of a gait analysis service.

## Methods

### Participants

For this empirical retrospective study, data collected during the clinical examination as part of the instrumented three-dimensional (3D) gait analysis were analyzed. Participants included 34 healthy, typically developing children aged 5–17 years, free of neuro-orthopedic diseases, or any other circumstances that could adversely affect their gait. This work was done in accordance with standard operating procedures of the gait lab and in compliance with ethical guidelines. Informed consent and assent were obtained from the parents of the children. Participants were allowed to leave the study at any point of time. Participants’ characteristics are shown in Table 1. Each clinical examination was carried out by the same team of physiotherapists and lab engineer, all experienced with clinical gait analysis.

**Table 1.** Demographic information of the 34 typical developing children displayed as mean (standard deviation).

	Total (*n* = 34)	Girls (*n* = 16)	Boys (*n* = 18)
Age (y)	10.9 (3.9)	12.1 (4.3)	9.9 (3.3)
Body height (m)	1.44 (0.20)	1.47 (0.19)	1.40 (0.20)
Body weight (kg)	37.9 (14.4)	38.9 (13.7)	37.0 (15.4)
BMI (kg/m^2^)	17.4 (2.7)	17.3 (2.2)	17.4 (3.2)

### Measurement methods

The neutral zero method^20^ was used to measure bilateral ROM of the following: hip extension and flexion, hip abduction and adduction, hip internal and external rotation in supine and prone position, knee extension and flexion, ankle plantarflexion, and dorsiflexion with reference to the hindfoot. This method is an accepted standard in clinical practice.^21^ For ROM measurement, one examiner positioned and stabilized the limb while a second examiner measured the joint angle in question by mean of a universal goniometer. Goniometers have been proven to be a valid and reliable method of measuring joint ROM in experienced examiners.^22^

As part of the clinical examination, muscle strength of the upper abdomen, iliopsoas, gluteus maximus, gluteus medius/minimus, hamstrings, and quadriceps femoris were tested bilaterally using MMT.^23^ Here, the examiner instructs the participant to assume a starting position depending on the muscle to be tested. The examiner then moves the participant’s joint actively to demonstrate the desired sequence of movement. The participant then performs the movement. After a few completed movement sequences, the assessor applies resistance to the movement with the hand. An isometric assessment of force is performed by applying an equal opposing torque.^24^ Muscle strength is assessed as a function of resistance applied and graded between 0 and 5 in steps of one, 0 being no strength, and 5 being maximum strength. Some grades are subdivided into half grades. The range of grades and their meanings are shown in Table 2.

**Table 2.** Medical Research Council scale modified according to Paternostro-Sluga et al.^14^ Selective Control Assessment of the Lower Extremity.^18^

MMT		SVMC	
Grade	Description	Grade	Description
0	No contraction	0 “pattern”	Desired movement sequence cannot be executed independently or a synergetic mass movement pattern according to Olree et al.^25^ can be seen
1	Flicker or trace contraction		
2	Active movement, with gravity eliminated		
2-3	Active movement against gravity over less than 50% of the feasible ROM		
3	Active movement against gravity over more than 50% of the feasible ROM		
3-4	Active movement against resistance over less than 50% of the feasible ROM	1 “partially”	Does not correspond to the passive ROM or if a movement can be observed in other joints
4	Active movement against resistance over more than 50% of the feasible ROM		
4-5	Active movement against strong resistance over the feasible ROM, but distinctly weaker than the contralateral side	2 “isolated”	Movement sequence takes place without movements of untested joints
5	Normal power		

To assess specific, isolated joint movements, the SVMC is evaluated based on the SCALE.^18^ The tested joint movements realized by the following muscles and muscle groups include the upper abdomen, iliopsoas, gluteus maximus, gluteus medius/minimus, hamstrings, and quadriceps femoris. The joint movement implemented by the tested muscle as well as the entire body is observed and evaluated using the SCALE with 0 “pattern,” 1 “partial,” or 2 “isolated.” The grades and their meanings are shown in Table 2.

**Figure 1.**
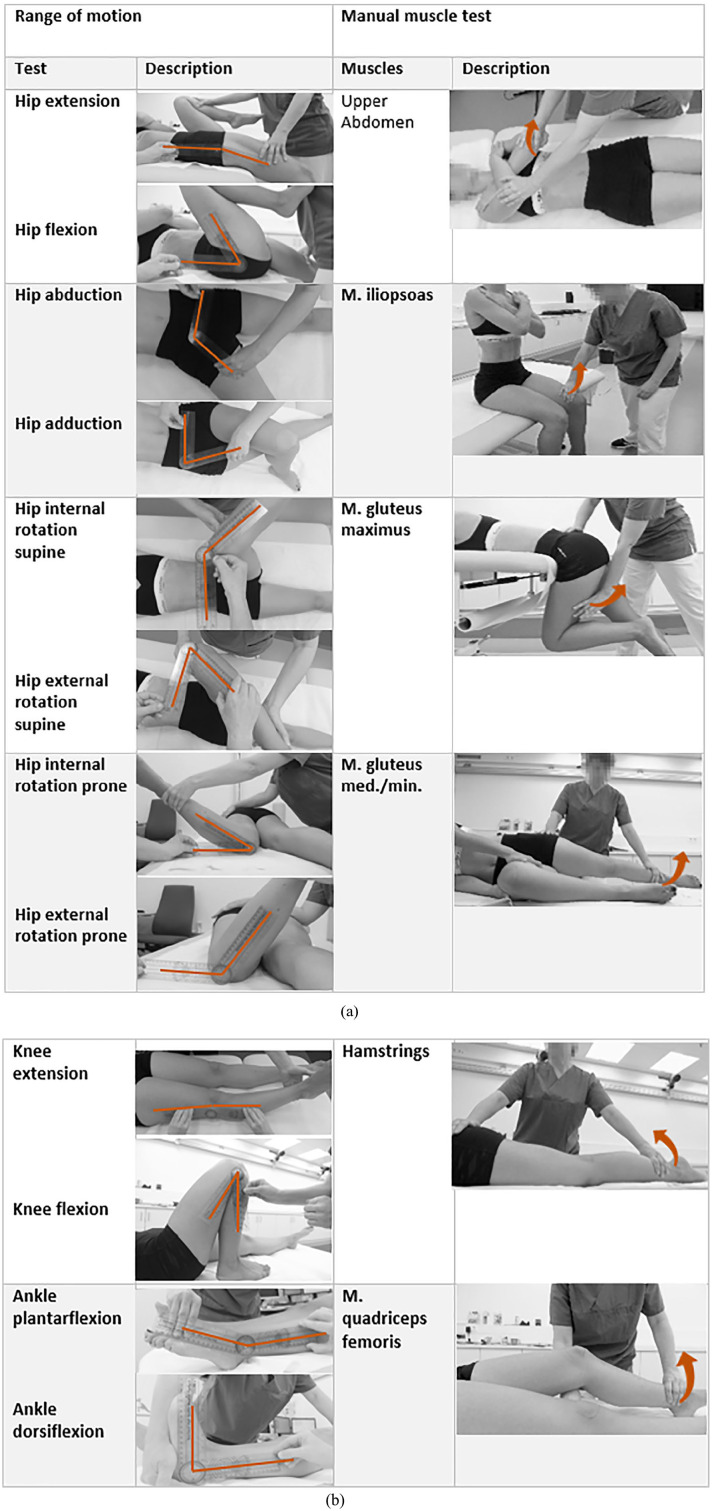
(a) Standardized measurement protocol. ROM measured in the neutral zero method and muscle strength per MMT for different muscles. The orange line in the ROM indicates the angle which is examined. The orange arrow in the MMT indicates the direction of the performed movement. The ROM test first measured movements away from the body and then movements toward the body in all planes of the body.^26^ (b) Standardized measurement protocol. ROM measured in the neutral zero method and muscle strength per MMT for different muscles. The orange line in the ROM indicates the angle which is examined. The orange arrow in the MMT indicates the direction of the performed movement. First, the movements away from the body and then the movements toward the body were measured in all planes of the body.^26^

The implementation of the MMT and the movement sequence for testing the SVMC for the individual muscles and muscle groups are illustrated in Figure 1. In addition, the functional HRT is performed to assess the strength of the ankle plantarflexors. Unlike the MMT, the resistance in this function test is not provided by the examiner, rather only by gravity and the participant’s own body weight.^27^ For a unilateral HRT, the examiner demonstrates the movement with slight stabilization of fingertips on the wall, bending the contralateral leg and fully extending the ipsilateral knee. She then raises the heel to maximum height and lowers slowly to the ground keeping the knee extended. The participant then performs the test. The maximum number of executions is noted with the test being aborted when compensation is visible. The standard for normal muscle function is 20 repetitions.^15,16^

### Data analysis

Microsoft Excel and MATLAB (MATLAB R2020b, The MathWorks, Inc., Natick, MA, USA) were used for the statistical analysis of the data. Left and right values were averaged for each participant for MMT, SVMC, and ROM. The average grades and angles of each participant are used to calculate the mean and standard deviation for the entire sample.

A one-sided *t*-test is used to examine whether the distribution of the grades differs statistically from the expected value of 5 on the mMRC scale or 2 on the SCALE with a probability of error of 5%. Mean values and standard deviations were calculated for all parameters. Significance level was set for *p* < 0.05.

## Results

### ROM

Hip flexion and extension, hip abduction and adduction, hip internal and external rotation in prone and supine position, knee flexion and extension, and ankle dorsiflexion and plantarflexion are presented in Table 3.

**Table 3.** Range of motion reference values of 34 typically developing children, displayed as mean in degree (standard deviation).

Measures of ROM	Entire sample degree (SD)
Hip extension (conventional Thomas Test)	9 (8)
Hip flexion (conventional Thomas Test)	139 (19)
Hip abduction (supine)	41 (10)
Hip adduction (supine)	20 (6)
Hip internal rotation (supine)	41 (15)
Hip external rotation (supine)	55 (12)
Hip internal rotation (prone)	50 (13)
Hip external rotation (prone)	32 (13)
Knee extension (supine)	4 (4)
Knee flexion (supine)	157 (4)
Ankle plantarflexion (knee extended)	34 (6)
Ankle dorsiflexion (knee extended)	20 (6)

### Muscle strength

**Figure 2.**
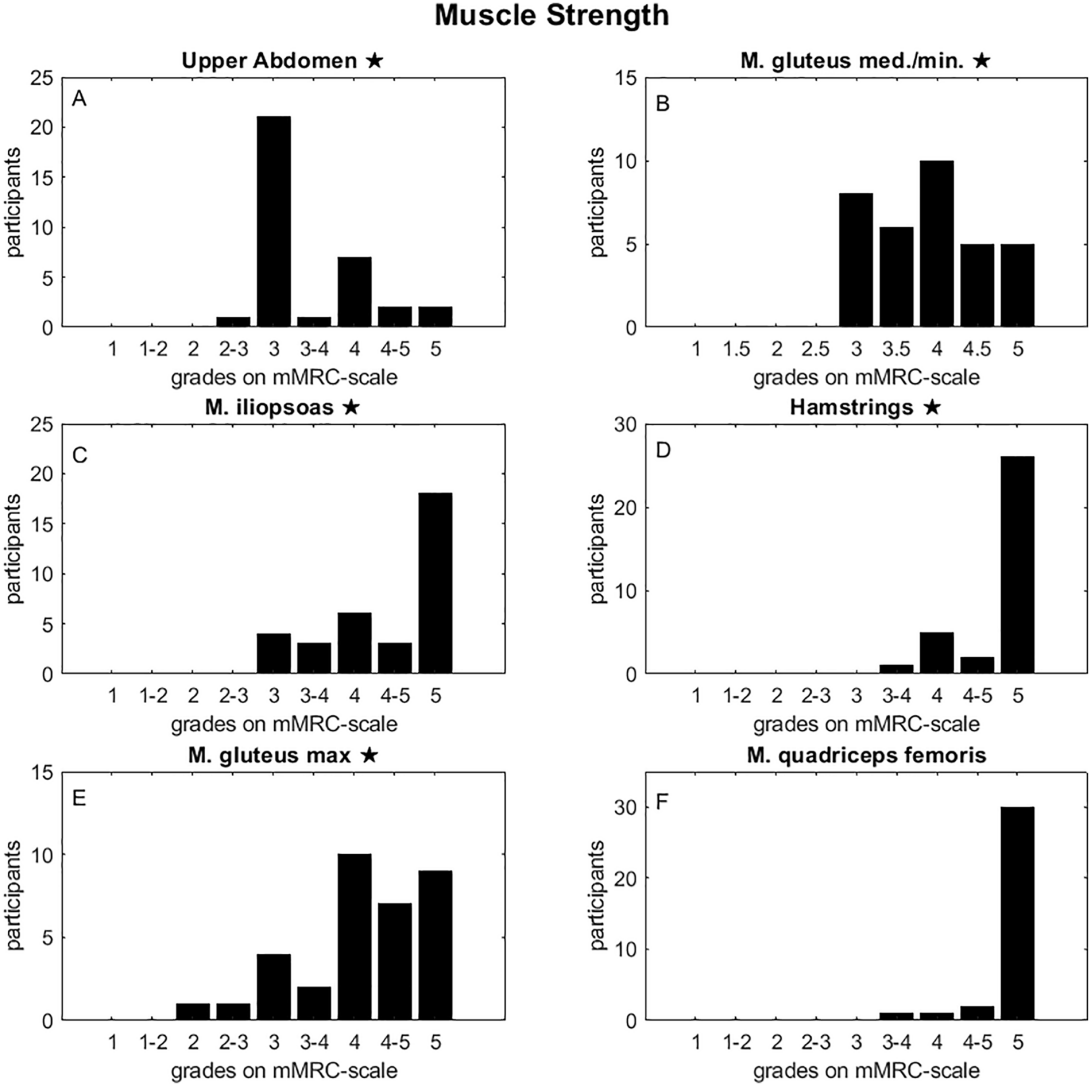
Muscle strength grades across the MMT. Muscle strength grades across the MMT for all participants assessed using the mMRC scale for the following muscles and muscle groups: upper abdomen (a), gluteus medius/minimus (b), iliopsoas (c), hamstrings (d), gluteus maximus (e), and quadriceps femoris (f). ★: Significant difference from maximum value 5 with a probability error of 5%.

Muscle strength and HRT were tested on 34 participants. Since the data were recorded in the context of daily clinical practice over an extended period that involved minor improvements with time and experience, the grades of the SVMC are incomplete. The number of available grades of the SVMC differ for the assessed joint movements and lie between 22 participants for M. quadriceps femoris and 32 participants for M. iliopsoas. The muscle strength grades for all participants assessed using the mMRC scale are shown in Figure 2.

An important finding is that not all typically developing children achieve a grade of 5 on the mMRSC scale in the manual strength test (MMT). There are also clear muscle-specific differences in the measured distribution (Figure 1). The hamstrings and the M. quadriceps femoris show a tendency toward particularly high scores of 5. According to the results of the one-sided *t*-test, apart from the quadriceps femoris muscle, all other distributions differ significantly from the maximum value of 5 on the mMRC scale with a probability of error of 5%. The mean number of repetitions of unilateral HRT which the typically developing children could perform is 12 ± 5.

### SCALE

**Figure 3.**
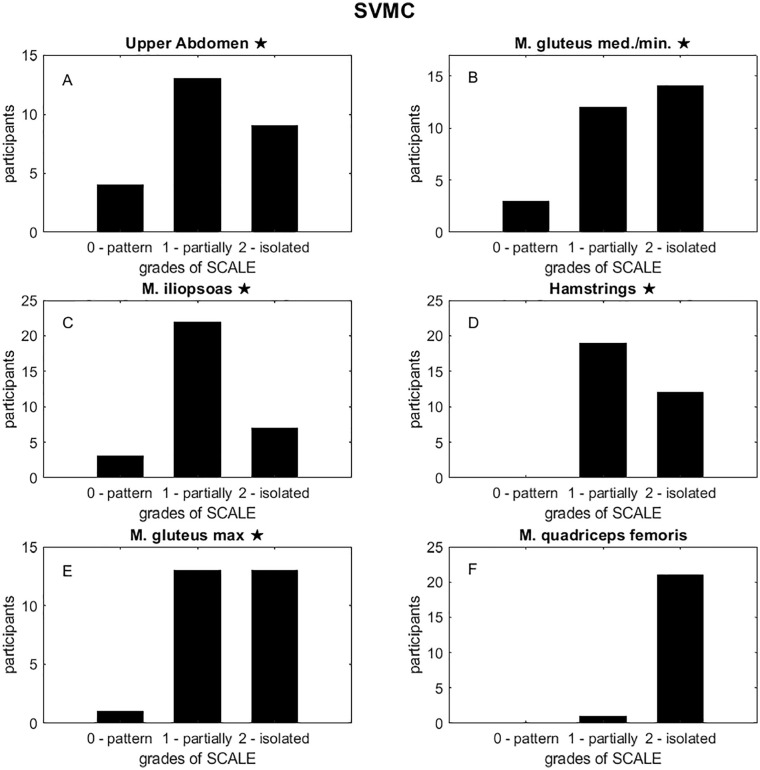
Grades of the SVMC. Grades of the SVMC for all participants evaluated using the SCALE for the joint movements realized by the following muscles and muscle groups: Upper abdomen (a), gluteus medius/minimus (b), iliopsoas (c), hamstrings (d), gluteus maximus (e), and M. quadriceps femoris (f). ★: Significant difference from maximum value 2 with a probability error of 5%.

The SCALE ratings of the SVMC for the entire sample are shown in Figure 3.

As with mMRC, the typically developing children did not always reach the maximum grade of 2 with SCALE. The grades in the evaluation of the SVMC are mainly in the upper and middle range. However, when testing the upper abdomen, the M. iliopsoas, the M. gluteus maximus, and the M. gluteus medius/minimus, there are few participants with the grade 0 “pattern.”

The results of the SVMC show that all distributions, with the exception the M. quadriceps femoris, differ significantly from the maximum value of 2 on the SCALE with a probability error of 5%.

## Discussion

The primary purpose of this study was to provide reference data regarding ROM, muscle strength, and SVMC for the lower limb in children in order to improve our understanding of these parameters and their variance both within and between muscles or joints.

Interestingly, typically developing children did not match the standard grades and repetitions in some examinations. This is of great importance in the assessment of typically developing and those with orthopedic or neuro-orthopedic conditions. Practice-relevant findings and correlations between muscle strength and SVMC can be derived.

In this study, ROM was measured for six different joint motions. Sankar et al.^10^ investigated ROM in the hip in typically developing children divided into three different age groups. The results of their study are in agreement with our study for hip abduction and adduction, as well as with the internal and external rotations in both positions. However, it is noticeable that the hip extension has clearly higher values for all three age groups compared to our study. This could be due to the execution of the measurement, as the hip extension was measured in the prone position in some studies, compared to our study where it was examined in the supine position. The knee flexion and extension is larger in our study than in other studies with typically developing children.^4,5^ McKay et al.^5^ reported a mean knee flexion of 140° degree for boys and 142° degree for girls in the age group of 10–19 years, which is slightly lower compared to our results (mean: 157°). The results of McKay et al.^5^ are, however, in alignment with the study of Soucie et al.,^4^ who also showed a lower degree for the knee flexion in children in the age of 9–19 years (mean: 142°). The studies on the joint angle for the knee extension are consistent with the ROM in our study.^3,4^ For the ankle dorsiflexion, our values are consistent with other available studies in typically developing children in similar age groups.^4,7^ However, the ankle plantarflexion shows noticeable differences in the ROM of our study, compared to other studies. While the studies from Soucie et al.^4^ and McKay et al.^5^ clearly indicate larger mean joint angles for plantarflexion of the foot in children (Soucie: 52°; McKay: 58° for boys, 62° for girls), the study of Alves et al.^28^ demonstrates a mean joint angle of 35°, which is in agreement with the 34° plantarflexion measured in the present study. The studies used the same measurement method, as well as typically developing children of similar age groups. Different ROM values between studies might be due to differences in extending forces applied by the study-specific examiner. The ROM depends on the passive joint characteristics and muscle properties as well as on the pain threshold of the participant. A reproducible and reliable determination of the ROM is only possible if joint-specific joint moments are specified.^29^

It is striking that, the best possible average grade of 5 on the mMRC scale was not achieved by typically developing children for all muscles and muscle groups, except for the ischiocrural musculature and the quadriceps femoris muscle. So far, no complete reference data set including typical developing participants and using the mMRC scale can be found for comparative purposes. There are only few studies in the literature that examined the muscle strength mostly with a handheld dynamometer.^5,12^ Bohannon^27^ measured the grades achieved on the MMT and the knee extensors forces via a handheld dynamometer. In agreement with our results, the adult participants receive an average grade of 4, and thus also do not necessarily achieve the maximum value on the rating scale. The fact that typically developing children do not reach grade 5 has numerous consequences for clinical investigations and research studies. Excessively high expectations lead to an erroneously underestimated assessment of typically developing children. Moreover, children with orthopedic or neuro-orthopedic disorders are rated even worse. This aspect should be considered by therapists and other medical practitioners. Furthermore, MMT grades rely largely upon the examiner’s judgment of the amount of force generated by the participant and, therefore, are subjective and prone to examiner bias. An alternative method is to perform the MMT additionally with a handheld or an isokinetic dynamometer.^30,31^ Unfortunately, due to physical and psychological constraints, this is hugely impractical, if not impossible to carry out on patients with neuro-orthopedic ailments, so that MMT and assessment using the mMRC scale is used.

The present sample achieves an average of 12 repetitions in the HRT. In comparison, Maurer et al.,^32^ in their study of 7- to 9-year-old children, report a much larger mean of up to 41.5 heel raises for 9-year-olds during HRT with their dominant leg. However, they also show very large standard deviation of 17.9 which could be explained by the relatively young age of the participants. The expected standard of 20 repetitions was reported by Jan in their study involving adult participants aged 21–40 years.^17^ They document a dependence between age and repetitions in HRT for adults. Although these two studies provide an initial reference data set, further research is needed to generate extensive reference data for all age groups and conditions. When assessing strength of the plantarflexors via the HRT, the 20 repetitions provided as a standard should be reconsidered and adjusted according to age. This work provides an initial reference data set for typically developing children that can be used for this purpose.

During execution of isolated joint movements participants achieved grades of either 1 “partially” or 2 “isolated” based on the SCALE. Up to now, there are no other studies that provide a reference data set for the assessment of SVMC using the SCALE either for children or for adults. From our results, we draw the conclusion that the grade of 2 “isolated” for SVMC on SCALE is also not a standard that can individually describe a typically developing child.

Kusumoto et al.^33^ studied the relation of lower extremity SVMC with knee extensor strength. They recognized a relationship of SVMC with maximum muscle strength of knee extensors. Balzer et al.^34^ described high correlations between MMT and SCALE grades. The correlation lies in the recruitment of motor units that can be influenced by impaired SVMC and significantly influence muscle strength.^33^ In children with spastic diplegia, there is sometimes no difference in maximum muscle strength between the affected and non-affected sides, although SVMC shows a difference.^33^ The reason for this is that spasticity in children with CP often manifests under load.^35^ This load is given by resistance when assessed via the MMT, unlike when SVMC is assessed without load. Accordingly, SVMC and muscle strength should be examined combined in the clinical examination and used dependently for interpretation.

### Limitations

This study focuses on typically developing children aged 5–17 years. During this time, there are great changes in mobility and muscle strength. This might yield to large variability in the measured parameters and may influence statistical outcome. Thus, future studies should examine smaller age groups and, if possible, investigate gender-specific differences. As suggested by Macfarlane et al.,^12^ the order in which the testing was carried out stayed the same for every participant. Due to the number of different tests, fatigue, concentration, or tiredness of the children could be a possible limitation, biasing the results. Because children vary in activity levels, the training status of children should be recorded to ensure comparability of data for children with motor impairments.

This study shows that typically developing children do not always achieve the assumed gold standards that are prescribed in the literature especially for muscle function tests. We recommend that caution be exercised when assessing children with orthopedic or neuro-orthopedic disabilities in the sense of adjusting our expectation based on the reference parameters provided here.

## Supplemental Material

sj-pdf-1-cho-10.1177_18632521241234768 – Supplemental material for Reference measures of lower-limb joint range of motion, muscle strength, and selective voluntary motor control of typically developing children aged 5–17 years Supplemental material, sj-pdf-1-cho-10.1177_18632521241234768 for Reference measures of lower-limb joint range of motion, muscle strength, and selective voluntary motor control of typically developing children aged 5–17 years by Emily Scherff, Sabrina Elisabeth Schnell, Tobias Siebert and Sonia D’Souza in Journal of Children’s Orthopaedics

## References

[1] Sarathy K Doshi C Aroojis A. Clinical examination of children with cerebral palsy. Indian J Orthop 2019; 53(1): 35–44. 30905980 10.4103/ortho.IJOrtho_409_17 PMC6394192

[2] Mudge AJ Bau KV Purcell LN , et al. Normative reference values for lower limb joint range, bone torsion, and alignment in children aged 4-16 years. J Pediatr Orthop B 2014; 23(1): 15–25. 23852035 10.1097/BPB.0b013e328364220a PMC13007707

[3] American Academy of Orthopaedic Surgeons. Joint motion: method of measuring and recording. Edinburgh: Churchill Livingstone, 1965.

[bibr4-18632521241234768] Soucie JM Wang C Forsyth A , et al. Range of motion measurements: reference values and a database for comparison studies. Haemophilia 2011; 17(3): 500–507. 21070485 10.1111/j.1365-2516.2010.02399.x

[bibr5-18632521241234768] McKay MJ Baldwin JN Ferreira P , et al. Normative reference values for strength and flexibility of 1,000 children and adults. Neurology 2017; 88(1): 36–43. 27881628 10.1212/WNL.0000000000003466 PMC5200854

[bibr6-18632521241234768] Ohman AM Beckung ER. Reference values for range of motion and muscle function of the neck in infants. Pediatr Phys Ther 2008; 20(1): 53–58. 18300934 10.1097/PEP.0b013e31815ebb27

[bibr7-18632521241234768] Alanen JT Levola JV Helenius HY , et al. Ankle joint complex mobility of children 7 to 14 years old. J Pediatr Orthop 2001; 21(6): 731–737. 11675545

[bibr8-18632521241234768] Da Paz SN Stalder A Berger S , et al. Range of motion of the upper extremity in a healthy pediatric population: introduction to normative data. Eur J Pediatr Surg 2016; 26(5): 454–461. 26393337 10.1055/s-0035-1563676

[bibr9-18632521241234768] Barad JH Kim RS Ebramzadeh E , et al. Range of motion of the healthy pediatric elbow: cross-sectional study of a large population. J Pediatr Orthop B 2013; 22(2): 117–122. 23238025 10.1097/BPB.0b013e32835c2be9

[bibr10-18632521241234768] Sankar WN Laird CT Baldwin KD. Hip range of motion in children: what is the norm? J Pediatr Orthop 2012; 32(4): 399–405. 22584842 10.1097/BPO.0b013e3182519683

[11] Bennell K Khan KM Matthews B , et al. Hip and ankle range of motion and hip muscle strength in young female ballet dancers and control. Br J Sports Med 1999; 33(5): 340–346. 10522638 10.1136/bjsm.33.5.340 PMC1756204

[12] Macfarlane TS Larson CA Stiller C. Lower extremity muscle strength in 6- to 8-year-old children using hand-held dynamometry. Pediatr Phys Ther 2008; 20(2): 128–136. 18480711 10.1097/PEP.0b013e318172432d

[13] Florence JM Pandya S King WM , et al. Intrarater reliability of manual muscle test (Medical Research Council scale) grades in Duchenne’s muscular dystrophy. Phys Ther 1992; 72(2): 115–122; discussion 122–126. 1549632 10.1093/ptj/72.2.115

[14] Paternostro-Sluga T Grim-Stieger M Posch M , et al. Reliability and validity of the Medical Research Council (MRC) scale and a modified scale for testing muscle strength in patients with radial palsy. J Rehabil Med 2008; 40(8): 665–671. 19020701 10.2340/16501977-0235

[15] Lunsford BR Perry J. The standing heel-rise test for ankle plantar flexion: criterion for normal. Phys Ther 1995; 75(8): 694–698. 7644573 10.1093/ptj/75.8.694

[16] Avers D Brown M. Daniels and Worthingham’s muscle testing: techniques of manual examination and performance testing. 10th ed. St. Louis, MO: Elsevier, 2019.

[17] Jan MH Chai HM Lin YF , et al. Effects of age and sex on the results of an ankle plantar-flexor manual muscle test. Phys Ther 2005; 85(10): 1078–1084. 16180956

[18] Fowler EG Staudt LA Greenberg MB , et al. Selective Control Assessment of the Lower Extremity (SCALE): development, validation, and interrater reliability of a clinical tool for patients with cerebral palsy. Dev Med Child Neurol 2009; 51(8): 607–614. 19220390 10.1111/j.1469-8749.2008.03186.x

[19] Fahr A Keller JW Balzer J , et al. Quantifying age-related differences in selective voluntary motor control in children and adolescents with three assessments. Hum Mov Sci 2021; 77: 102790. 33798928 10.1016/j.humov.2021.102790

[20] Ryf C Weymann A. The neutral zero method—a principle of measuring joint function. Injury 1995; 26(1): 1–11.

[21] Schiefer C Kraus T Ellegast RP , et al. A technical support tool for joint range of motion determination in functional diagnostics—an inter-rater study. J Occup Med Toxicol 2015; 10: 16. 25983852 10.1186/s12995-015-0058-5 PMC4433057

[22] Gogia PP Braatz JH Rose SJ , et al. Reliability and validity of goniometric measurements at the knee. Phys Ther 1987; 67(2): 192–195. 3809242 10.1093/ptj/67.2.192

[23] Kendall FP McCreary EK. Muskeln—Funktionen und Test. Stuttgart: Gustav Fischer Verlag, 1988.

[24] Lewinson RT Ganesh A Yeung MMC . The biomechanics of manual muscle testing in the neuromuscular exam. Can J Neurol Sci 2018; 45(5): 518–521. 30071911 10.1017/cjn.2018.53

[25] Olree KS Engsberg JR Ross SA , et al. Changes in synergistic movement patterns after selective dorsal rhizotomy. Dev Med Child Neurol 2000; 42(5): 297–303. 10855649 10.1017/s0012162200000530

[26] Debrunner HU and Schweizerische Arbeitsgemeinschaft für Osteosynthesefragen. Gelenkmessung: (Neutral-O-Methode), Längenmessung, Umfangmessung. Bern: Schweizerische Arbeitsgemeinschaft für Osteosynthesefragen, 1971.

[27] Bohannon RW. Quantitative testing of muscle strength: issues and practical options for the geriatric population. Top Geriatr Rehabil 2002; 18(2): 1–17.

[28] Alves C Lysenko M Tomlinson GA , et al. Plantar flexion, dorsiflexion, range of movement and hindfoot deviation are important determinants of foot function in children. J Child Orthop 2019; 13(5): 486–499. 31695816 10.1302/1863-2548.13.190062 PMC6808068

[29] Siebert T Donath L Borsdorf M , et al. Effect of static stretching, dynamic stretching, and myofascial foam rolling on range of motion during hip flexion: a randomized crossover trial. J Strength Cond Res 2022; 36(3): 680–685. 34379375 10.1519/JSC.0000000000003517

[30] Siebert T Kurch D Blickhan R , et al. Does weightlifting increase residual force enhancement? J Biomech 2016; 49(10): 2047–2052. 27234620 10.1016/j.jbiomech.2016.05.017

[31] Holzer D Paternoster FK Hahn D , et al. Considerations on the human Achilles tendon moment arm for in vivo triceps surae muscle-tendon unit force estimates. Sci Rep 2020; 10(1): 19559. 33177655 10.1038/s41598-020-76625-x PMC7658232

[32] Maurer C Finley A Martel J , et al. Ankle plantarflexor strength and endurance in 7–9 year old children as measured by the standing single leg heel-rise test. Phys Occup Ther Pediatr 2007; 27(3): 37–54. 17613455

[33] Kusumoto Y Takaki K Matsuda T , et al. Relation of selective voluntary motor control of the lower extremity and extensor strength of the knee joint in children with spastic diplegia. J Phys Ther Sci 2016; 28(6): 1868–1871. 27390436 10.1589/jpts.28.1868 PMC4932077

[34] Balzer J Marsico P Mitteregger E , et al. Influence of trunk control and lower extremity impairments on gait capacity in children with cerebral palsy. Disabil Rehabil 2018; 40(26): 3164–3170. 28944697 10.1080/09638288.2017.1380719

[35] Ubhi T Bhakta BB Ives HL , et al. Randomised double blind placebo controlled trial of the effect of botulinum toxin on walking in cerebral palsy. Arch Dis Child 2000; 83(6): 481–487. 11087280 10.1136/adc.83.6.481 PMC1718586

